# Direct method for Ra-226 analysis in water samples using ICP-QQQ-MS

**DOI:** 10.1038/s41598-025-18775-4

**Published:** 2026-01-22

**Authors:** Ivana Coha, Marko Štrok, Norbert Kavasi

**Affiliations:** 1 Department of Environmental Sciences, Jožef Stefan Institute, 1000 Ljubljana, Slovenia; 2 Laboratory for Radioecology, Division for Marine and Environmental Research, Ruđer Bošković Institute, 10000 Zagreb, Croatia; 3 Regional Environmental Co-Creation Unit, Fukushima Institute for Research, Education and Innovation, Fukushima, 960-1295 Japan; 4 Department of Biophysics and Radiation Biology, Semmelweis University, 1085 Budapest, Hungary

**Keywords:** Ra-226, Water sample, Mass spectrometry, Standard addition, IAEA proficiency test, Environmental monitoring, Mass spectrometry

## Abstract

The paper describes a method of determination of Ra-226 in natural water using ICP-QQQ-MS, Agilent 8900, that enables an efficient direct determination of Ra-226 with high sensitivity without using any reaction gas and separation procedures for interference removal. The matrix effect, together with the instability of the sensitivity are controlled by spiking the sample with Ra-226. The method is simple and fast with a detection limit around of 0.1 fg g^-1^ (3.6 mBq kg^-1^), which is tenfold lower than required by the EU Directive for water consumption (40 mBq kg^-1^) and five times better than results obtained from alternative direct method for routine analysis described in the literature. The method can easily be implemented for routine analysis Ra-226 which was demonstrated by direct determination of Ra-226 in IAEA proficiency test water sample. This is the first time that the accuracy (trueness and precision) of a mass spectrometry method for the direct determination of Ra-226 has been tested and confirmed in an independent proficiency test.

**Supplementary Information:**

The online version contains supplementary material available at 10.1038/s41598-025-18775-4.

## Introduction

Naturally occurring radionuclides are typically remnants from the original formation of the Earth and are part of natural radioactive decay series. One of the most toxic natural radionuclides is Ra-226^1^, along with its descendants radon, lead and polonium. Ra-226 is a divalent alkaline earth metal and behaves chemically similarly to calcium. It can form soluble complexes, making it more mobile in water. This pathway can introduce Ra-226 in food chain and once it enters to human body, it can deposit in bones and teeth, thus increasing the internal radiation dose for individuals^2^. Additionally, a significant association was identified between exposure to radium in drinking water and an increased risk of colorectal cancer^3^.

Monitoring and managing radium levels are essential to ensure that its content in water sources comply with safety standards and guidelines^4,5^, protecting both human health and the environment, especially in areas with potential sources of contamination or where geochemical conditions are conducive to its mobilisation^6,7^.

Ra-226 is usually determined by radiometric methods; alpha/gamma spectrometry or by liquid scintillation counting (LSC)^8–10^. Its determination methodology requires preconcentration, separation, and purification procedures before detection and quantification^10–17^ which are often tedious and time consuming.

Mass spectrometry (MS) methods are viable alternative to radiometric methods, particularly for radionuclides determination with a half-life exceeding 100 years^18,19^. Ra-226 has been determined by several mass spectrometry techniques, such as accelerated mass spectrometry (AMS)^20^, thermal ionization mass spectrometry (TIMS)^21,22^ or most widely inductively coupled plasma mass spectrometry (ICP-MS); high resolution ICP-MS^23^, double-focusing sector field ICP-MS^15,24–28^ or multi collector ICP-MS^29^, collision-cell ICP-MS^30^ or most recently, triple quadrupole (QQQ)^31–34^. The advantage of MS is necessity of much smaller sample size, shorter analytical time, less need of chemical consumption, higher sample throughput and improvement in analytical precision^11,35–38^.

Although mass spectrometry (MS) has been employed in radionuclide determination for decades, its routine application remains limited in comparison to radiometric techniques. This underutilization can be attributed to several factors. Most notably, MS instrumentation is considerably more expensive and maintenance-intensive than radiometric systems, particularly when assessed in terms of achievable detection limits (e.g. AMS and LSC).

Furthermore, MS techniques such as inductively coupled plasma mass spectrometry (ICP-MS), thermal ionization mass spectrometry (TIMS), and multi-collector ICP-MS (MC-ICP-MS) require a higher level of operational expertise. Basically, the use of MS in the analysis of radionuclides was perceived as a “state of the art” technique that was reserved for the so-called “analysis of exotic samples” such as in nuclear forensics, extra-terrestrial/lunar samples, etc^39,40^. In recent years, however, the scope of MS in radionuclide analysis has broadened, particularly with its application in environmental, food, and water sample analysis. Nevertheless, these applications are predominantly research-oriented and have not yet transitioned into routine monitoring frameworks. The emergence of more cost-effective quadrupole ICP-MS instruments, which offer sufficient sensitivity and precision to meet regulatory thresholds, has begun to change this dynamic. Despite these advancements, routine analyses of radionuclides by ICP-MS, especially in natural water matrices, remain rare. Key limitations are high acquisition and operational costs of the instruments and often they are inaccessible to a wide range of users. For example, MC-ICP-MS is well known for its ability to measure isotopic ratios with extremely high precision, but the design of MC-ICP-MS for high-precision isotopic ratio measurement has a trade-off in sensitivity, which leads to increased detection limits for radionuclide measurements that are on the order of several ng/mL^37^. In this context, recent developments in quadrupole ICP-QQQ-MS technology, due to its potential for efficient spectral separation without sacrificing sensitivity and the ability to measure isotopic ratios with solid precision, seems particularly suitable for radionuclide measurements.

The accurate measurement of radionuclides by ICP-QQQ-MS, and thus Ra-226, is also impacted by multiple interferences. Ra-226 does not have stable isobaric interferences, but the formation of polyatomic interferences (e.g. ^88^Sr^138^Ba, ^87^Sr^139^La, ^86^Sr^140^Ce, ^40^Ar^40^Ar^146^Nd, ^18^O^208^Pb and several combinations of molybdenum isotopes with xenon isotope) or multiple charged ions, can contribute to the signal measured at m/z of 226 and result in overestimation of Ra-226 concentrations^23,27,28,30–32,41–43^. Conversely, it has also been shown that high concentrations of Ca, Mg and associated atoms in the sample matrix reduce the sensitivity of ICP-MS by impairing the ionization efficiency of the plasma or reduced ion transmission through gradual blocking of the interface cones. This can, therefore, lead to underestimated results^16,35,44,45^. In other words, accurate and reliable determination of Ra-226 requires to ensure the sufficient concentration of Ra-226 to reach the limit of quantification for the available instrument and removal of all interferences that could elevate that limit. These two conflicting requirements are generally managed through using separation and preconcentration procedures, most commonly involving cation exchange resin^15,16,26,32,45–47^, MnO_2_ resin^24,48^ or highly specific extraction chromatography resins such as TK100^43,49^, AnaLig®Ra-01^31^ or Ra-specific disks^50^. However, preconcentration and separation prolong the analysis time and additionally increase costs. In order to avoid these steps, efforts are being made to develop direct methods that either eliminate the need for these steps or minimize their use. One such method for the determination of Ra-226 in water has recently been described in the literature^51^. A method enables direct measurements of Ra-226 in water samples with ICP-QQQ-MS by using of N_2_O as reaction gas to ensure that no separation before analysis was necessary. The limit of detection of proposed method compliant with the specifications for methods used for routine analysis of drinking water quality according to European and U.S. regulations.

Considering that the concentration of Ra-226 and other dissolved substances in natural waters can vary in a wide concentration range from ng/kg to mg/kg, the main objective of this work is to develop a rapid and simple method for the direct determination of Ra-226 at the femtogram level in natural waters with different content of total dissolved substances without using separation/preconcentration procedures or reaction gas. The new generation of ICP-QQQ-MS, Agilent 8900, offers an increased matrix tolerance of up to 25% of total dissolved solids (TDS) compared to its predecessor 8800^52^ due to the aerosol dilution technique developed by Agilent. In contrast to the mentioned determination method, where N_2_0 was used as a reaction gas to remove interferences and lower the detection limits, this paper describes a standard addition method that allows an equally efficient direct determination of Ra-226 with improved sensitivity. It is known that the use of a reaction gas to eliminate interferences leads to a significant reduction in sensitivity^53^. Therefore, this paper presents how the use of the standard addition method with matrix matching can eliminate the effects of interferences and maximize the sensitivity of the above instrument. Particular attention was given to investigating the influence of matrix composition and Ra-226 concentration on detection limits, with the aim of maximizing sensitivity under the given conditions. The advantages and limitations of the proposed determination method were also discussed, with the goal of developing an affordable and user-friendly approach that enables wider application.

## Results and discussion

### Instrumental sensitivity for Ra-226 measurement

In the Table 1 comparison of instrumental sensitivity and instrumental detection limits for different ICP-MS instruments is given. The determined blank signal within this study on Agilent 8900 at 226 m/z mass to charge was generally very low, within a range from 0.08 to 0.16 cps. The instrumental Ra detection limit (IDL), defined as average of blanks plus 3 *SD*_blank_ divided by the slope of the calibration curve was always below 0.1 fg g^-1^ (3.6 mBq kg^-1^). As can be seen, the detection limit achieved in this work is comparable to the iCAP-Q ICP-MS coupled with Apex^16^ and one of the lowest achieved with ICP-QQQ-MS. In comparison with its predecessor, Agilent 8800, the detection limit achieved in a single quad mode is 4 to 5 times lower^32,49^.

**Table 1:** Comparison table of instrumental sensitivity and limits of detection for Ra-226 by ICP-MS.

Reference	Model of the instrument	Nebulizer, special introduction system	Instrumental sensitivity in cps for 1 pg g^-1^	Instrumental detection limit		Operation model
				fg g^-1^	mBq kg^-1^	
This work	Agilent 8900	MicroMist nebulizer	2700	0.1	3.6	MS
Bonin^33^	Agilent 8900	MicroMist nebuliser and double-pass spray chamber	2800	0.05	2	MS
Waersted^51^	Agilent 8900	MicroMist nebulizer	N/A	0.42	15.4	MS/MS + N_2_O
Van Es^49^	Agilent 8800	MicroMist nebulizer	330	0.420	15	MS
		MicroMist nebulizer	168	N/A	N/A	MS/MS
Dalencourt^32^	Agilent 8800	N/A	176	0.5	18.3	MS
		N/A	17	8	292.8	MS/MS
		N/A	9	14	512.4	MS/MS + ORS (1 ml min^-1^ He)
Lagace^16^	Thermo Scientific iCAP-Q	APEX-Q	N/A	0.2	7.3	MS
Zhang^45^	NexION 300x	N/A	77.6	100 ± 45 (% RSD)	3660	Standard mode
		N/A	34.44	100 ± 67 (% RSD)	3660	Collision mode using a He gas and kinetic energy discrimination (KED)
Verlinde^31^	Agilent 8800	Scott spray chamber	329.4	1.86	68	MS
		APEX HF with PFA microFlow nebulizer	10 × higher than with the Scott spray chamber	0.22	8	MS
	Thermo scientific iCAP-Q	APEX Q with a membrane desolvation module	10 × higher than with the Scott chamber	0.16	6	MS
	Element 2	APEX Q with a membrane desolvation module	~ 80 × higher than with the Scott chamber	0.03	1	HR-MS
Amr^48^	Agilent, 7500Ce	ARDIUS, CETAC, USA	N/A	11.3	414	MS + He gas (1 ml/min He)

The sensitivity of Agilent 8900 is even greater and similar to one obtained in a different study^33^, which is in the same range as the sensitivity achieved by combination of Agilent 8800 with a dedicated sample introduction system like Apex^31^. Specialised desolvating nebulisers, such as Apex or Aridus, have been regularly used to improve sensitivity and lower detection limits. However, Ra-226 must be separated from the matrix prior to analysis to prevent clogging of the desolvating nebulizer, thus direct measurement in water samples is not feasible nor recommended. The high sensitivity of Agilent 8900 can be sufficient for developing a direct and fast method for Ra-226 monitoring in water samples. It is achieved due to enhanced ion transmission by a new vacuum stage at the interface.

### Interference and matrix effect tests

ICP-QQQ-MS offers the possibility of eliminating possible polyatomic interferences and matrix effect by combining two quadrupoles and a collision/reaction cell. However, this also affects the sensitivity of the device, so that the instrumental detection limit increases. When using the MS/MS mode (using both quadrupoles) with He as the collision gas, the sensitivity decreased almost fourfold. Dalencourt^32^ has shown that on Agilent 8800 the instrumental detection limit is 0.5 fg g^-1^ in MS mode, 8 fg g^-1^ in MS/MS mode and 14 fg g^-1^ in MS/MS with octopole reaction system (ORS) as a collision cell mode, which is almost 30 times higher than in MS mode. In recently developed direct method to determine Ra using the MS/MS mode with N_2_O as the reaction gas, detection limit achieved by Agilent 8900 was 0.42 fg g^-1^ (15 mBq kg^-1^) for water samples^51^. Although this approach helped to eliminate interferences, the detection limit was increased compared to using a single quadrupole and without reaction gas. As the 8900 has an improved sensitivity and higher tolerance to matrix, further experiments focused on optimising the conditions for direct water sample analysis using single quad mode without reaction gas.

**Fig. 1:**
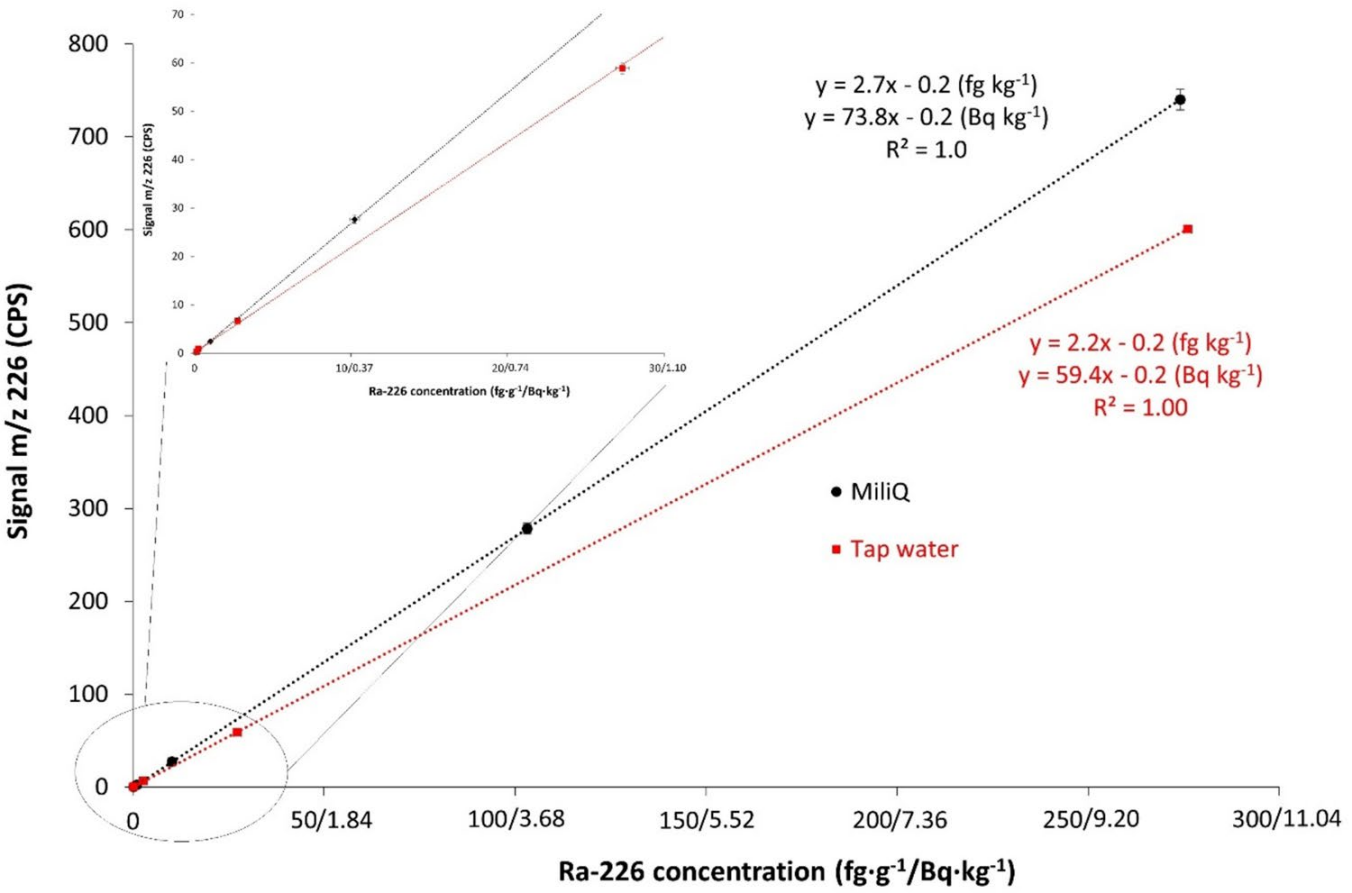
Calibration curves for Ra-226 standards (0.1—275 fg g^-1^/0.004—10.1 Bq kg^-1^) on Agilent 8900 ICP-MS in 2% nitric acid solution and tap water (2% nitric acid).

In Fig. 1 calibration curves for Ra-226 standards in 2% nitric acid solution and tap water acidified to 2% nitric acid solutions are compared. The slopes of the calibration curves show that the sensitivity in tap water is 20% lower than in 2% nitric acid solution. For the same matrix, however, the signal suppression is constant, as evidenced by a correlation coefficient of 1 (*R*^2^ = 1.0).

**Fig. 2:**
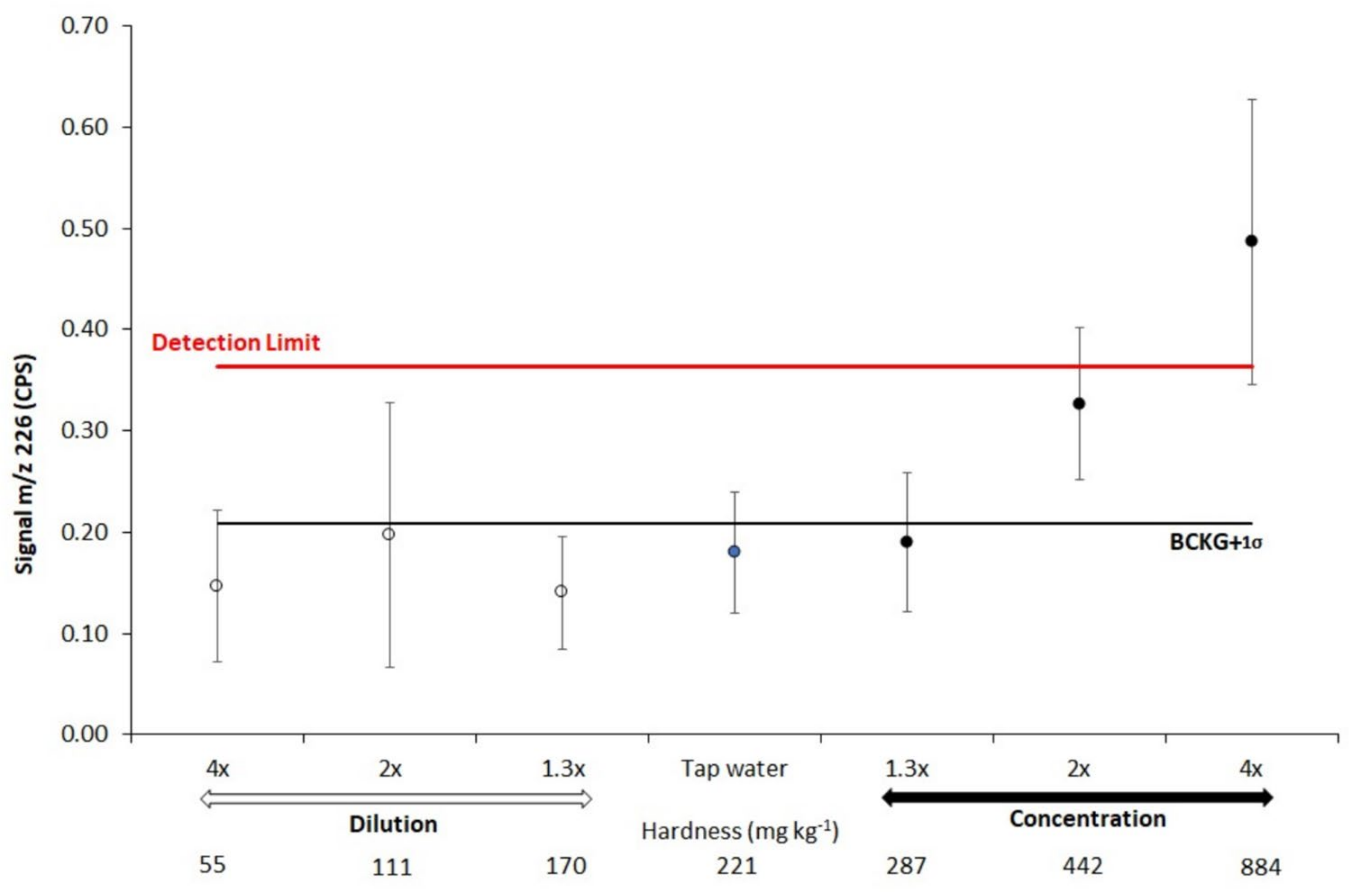
Signal intensity at m/z 226 in diluted and concentrated tap water.

In order to establish a direct analytical method, it is important to determine the interference effect at m/z 226 in the water sample. For this purpose, the signal at m/z 226 was determined in diluted and concentrated tap water samples (Fig. 2), where water hardness was from soft (55 mg kg^-1^) and moderately hard water (111 mg kg^-1^) to hard (170–287 mg kg^-1^) and very hard water (442 and 884 mg kg^-1^). It was found that in diluted samples the signal was identical to the background and below 1 sigma of the instrumental detection limit, while in concentrated samples (two and fourfold concentrations) the signal gradually increased above the IDL.

There are two possibilities to consider for fourfold concentrated water sample: (A) The observed signal at m/z 226 is ascribed to Ra-226, resulting from a fourfold concentration increase through evaporation, or (B) The signal at m/z 226 is caused by interference, as the concentrations of major and trace elements have been elevated. The significance of this test results lies in its demonstration that interference can be disregarded when the concentration of both main and trace elements falls within the values specified in Table S2. Interference may occur above this range of inorganic elements, leading to a spurious signal of Ra-226 in the range of a few mBq·kg^-1^. However, hypothesis A may be more realistic since when measuring by ICP-QQQ-MS, no increase of the signal was found when the concentration of Sr, Ba or Ca was below 10 µg g^-1^ per each element^53^. Additionally, from our experiments it was observed that mix of Sr and Ba at concentrations up to 2 µg g^−1^ Sr and 100 ng g^-1^ Ba in a solution did not increase the background at m/z = 226 in a Single Quad mode nor Pb concentration up to 100 ng g^-1^. It should be emphasized that the concentration of major and trace elements in the measured tap water samples was in µg g^-1^ and ng g^-1^ levels, respectively, while the range of Ra-226 concentration in tap water and spiked tap water up to 276 fg g^-1^ (10.1 Bq kg^-1^) was still 10^6^ up to 10^9^ lower than the concentration of Sr and Ba. In all samples measured, the background counts for the m/z 226 signal were very low (< 0.16 cps) and the Ra-226 m/z signal was constantly lower than in -nitric acid solution. These results indicate that there is no signal enhancement by polyatomic or multiple charged elements interferences in tap water, but rather signal suppression by the matrix effect. However, since different samples contain varying and unpredictable amounts of potential interferences, preparing a spiked sample in parallel and ensuring exact matrix match can help overcome this problem.

### Spike selection

The direct method was further developed primarily to enhance sensitivity and detection limits. Another important reason was the challenge of finding a suitable tracer for Ra-226 quantification if any separation or preconcentration of the sample is performed to further lower the detection limit. Ra-228, which is the longest-lived Ra (radio) nuclide after Ra-226, could be used as the spike, or as the internal standard for the direct method. However, the certified reference solution of Ra-228 contains Ra-226 which exact value is not given in the certificate, rather its estimated amount of less than < 0.5%. Additionally, attempts were made to produce pure Ra-228 by isolating it from purified Th-232. However, thorium nitrate contains a small amount of uranium and thus Th-230, from which Ra-226 is again generated. Therefore, pure Ra-228 without any significant contribution from Ra-226 could not be obtained. When detectable amounts of Ra-228 were isolated and measured, the activity of Ra-226 was significantly higher than its concentration in the unknown sample. Additionally, the determination of the exact Ra-228/Ra-226 isotopic ratio was beyond the scope of this work; therefore, a certified reference solution of Ra-226 was used for the standard addition method.

### Direct determination of Ra-226 by ICP-QQQ-MS

The instrumental sensitivity of the Agilent 8900 is several times higher than for any previously used instrument. This enabled the development of a method for the direct determination of Ra-226 without prior preconcentration or purification/separation, through the application of the standard addition method.

The proposed method, the direct determination of Ra-226 with standard addition, consist of:

1. Tuning the instrument
2. Element specific (Ra-226) tuning the instrument
3. Background measurement of- 2% nitric acid blank solution
4. Batch 1: Measurement of unspiked samples (preliminary measurement)
5. Batch 2: Measurement of unspiked and spiked samples

For direct water samples analysis, it is recommended to condition the cones by running some acidified tap water samples (without using the data) before the actual measurement since this step can help to minimize the drift in sensitivity. Additional stability of sensitivity was achieved by using acidified tap water or a synthetic water sample between the measured samples instead of a 2% nitric acid rinse solution. In the proposed way, the signal for the 10 mBq g^-1^ (274 pg kg^-1^) solution, was suppressed compared to the model standard solution, but was more stable throughout the working day. To estimate the amount of added Ra-226 spike for each sample, a preliminary measurement was carried out (Batch 1) before the final measurement (Batch 2). Thus, to determine Ra-226 in a water sample, the sample was measured three times (twice in the non-spiked sample, and once in the spiked sample). The detection limit for each sample was calculated based on the results of the unspiked and spiked samples as explained in the Supporting Information (S5). A practical example for Batch 2 is also included in the Supporting Information (S6).

### Method validation

To validate this method, different water samples (the 2% nitric acid solution, the acidified tap water sample (2% nitric acid) and QC sample from IAEA proficiency test) with added certified Ra-226 spike solution (CRM) were analysed. The results are presented in Table 2.

**Table 2:** Results of Ra-226 determination in various spiked water samples (IAEA QC, tap water sample and MQ water).

Sample code	Assigned value, Ra − 226 fg·g^−1^ (Bq·kg^−1^)	Measured value, Ra − 226 fg·g^−1^ (Bq·kg^−1^) (*N* = 5)	Ratio of (activity) concentration in the sample and the spike	Bias (%)	*Zeta* score**	Detection limit fg·g^−1^ (mBq·kg^−1^)***
MQ − 0.05	1.43 ± 0.01 (0.052 ± 0.001)	1.54 ± 0.38 (0.057 ± 0.014)	1:1	8.2	0.31	0.11 (4.2)
MQ − 0.1	2.86 ± 0.02 (0.105 ± 0.001)	2.64 ± 0.29 (0.097 ± 0.010)	1:4	− 7.6	− 0.76	0.10 (3.7)
MQ − 2.0	5.35 ± 0.04 (0.196 ± 0.001)	5.59 ± 0.68 (0.205 ± 0.025)	1:1	4.6	0.36	0.17 (6.2)
Mq − 0.5	14.4 ± 0.1 (0.53 ± 0.01)	14.9 ± 2.1 (0.54 ± 0.08)	1:1	3.0	0.20	0.11 (3.9)
Mq − 0.5	14.4 ± 0.1 (0.53 ± 0.01)	14.5 ± 0.9 (0.53 ± 0.04)	1:9	0.5	0.09	0.10 (3.8)
MQ − 1.0	107.4 ± 0.7 (3.93 ± 0.03)	112.6 ± 6.0 (4.12 ± 0.22)	1:1	4.8	0.86	0.11 (4.0)
QC − 23 − 1	60.11 ± 0.4 (2.20 ± 0.02)	58.8 ± 4.7 (2.15 ± 0.17)	1:2	− 0.3	− 1.45	0.24 (8.7)
QC − 23 − 2	165.7 ± 1.0 (6.07 ± 0.04)	174.9 ± 9.0 (6.40 ± 0.33)	1:1	5.5	1.01	0.19 (7.0)
TW	49.7 ± 0.3 (1.82 ± 0.01)	48.3 ± 4.5 (1.77 ± 0.17)	1:1	− 2.9	− 0.32	0.19 (7.1)

* Standard uncertainty with a coverage factor of k = *1.*

**If |Zeta|< *2 there is no significant difference at significance level of 5%*

***Detection limit calculation in the Supporting info (S5).

A good agreement between the expected and the determined Ra-226 activity concentration was obtained for all prepared samples. The variation of the deviation of the measured values from the expected values (relative bias in the range of -7.6% to 8.2%) shows that there is no systematic error and that the results obtained have a satisfactory accuracy. In addition, the zeta score is less than 2 in all cases, meaning that there is no significant difference between the measurement results and the assigned values. Accuracy, trueness and precision (including uncertainty) are satisfactory.

The uncertainty budget was calculated as described in the Supporting Information (S4). The relative uncertainty of all results was below 10% (*k* = 1) for samples with high activity (when the activity concentration of Ra-226 was above 1 Bq kg^-1^/27 fg g^-1^). For samples with lower activity concentrations, between 50 mBq kg^-1^/1.5 fg g^-1^ and 500 mBq kg^-1^/15 fg g^-1^, the uncertainties were between 6 and 24% (*k* = 1).

In the proposed protocol, unknown samples should be analysed first to determine the proportion of the standard (spike) addition. In the example shown for an activity concentration slightly above the permissible value for Ra-226 in drinking water, the uncertainty of the measured value decreased from 14 to 6% when a higher proportion of spike was added (analyte to spike concentration ratio 1:1 and 1:9). This is due to the fact that the uncertainty in the counting of the spiked samples has a high contribution to the total uncertainty budget. It is advisable to add a higher concentration of Ra-226 spike to the analyte to obtain better counting statistics and precision. Additionally, the counts derived from the spike should be several times higher than the deviation of the sample counts. However, the standard deviation of counts from the spiked sample should be lower than the number of counts from the unknown sample. In other words, the spike concentration should be chosen so that it results in measurable changes in the analyte signal without causing excessive variability. From the obtained measurement results and further data analysis the recommended ratio between analyte and spike concentration is at least 1:2 in the mBq·kg^-1^ range and at least 2:1 in Bq·kg^-1^ range.

Depending on the matrix, the suppression of the signal varies from sample to sample. This also has an influence on the detection limit. Although the detection limits of the proposed method correspond to the average activity concentration of Ra-226 in seawater and are higher than the average values in tap water, it can be used for natural water samples (especially mineral and thermal waters), treated water or for screening the activity level of water intended for human consumption. An additional reduction of the detection limits can be achieved simply by reducing the volume of the water sample by a factor of 10, e.g. by evaporation from 60 to 6 ml, with one subsample measured directly and one with spike addition.

The water samples are usually filtered and acidified to pH 2 during sampling. This means that the proposed method requires very simple handling prior to the measurements. Apart from acidification to a very weak acid solution (2% nitric acid) and spiking with Ra-226, no further measures need to be taken before the measurement. Due to the absence of element separation, the use of reagents is minimized, aligning with the principles of green laboratory practices. It is also possible to perform the spiking automatically if another autosampler is used. It takes less than 5 min to measure one sample, which means that a large number of samples (over 30) can be measured in a day. Due to its simplicity and high sample throughput, the proposed method is superior to any conventional radiometric methods or alternative ways for the determination of Ra-226 with separation by ICP-MS. For comparison, the most sensitive radiometric technique, alpha spectrometry, requires approximately 8 h for the rapid determination of Ra-226 in water samples, along with a significantly more extensive preparation process^54^.

### Confirmation by independent proficiency test exercise

The method presented here was independently tested as part of a proficiency test exercise organized by the IAEA laboratory. Together with the participants that are ALMERA laboratories and those that form a worldwide group, about 450 laboratories participated. The result for Ra-226 in water sample No. 2 was reported by 156 laboratories^55,56^. This means that 48% of the ALMERA laboratories and 67% of the participants from worldwide group did not report the result. Of the participants from the ALMERA laboratories, 34% reported acceptable results, while only 19% of the results from worldwide group were accepted. This confirms the need to develop simple and easy to implement methods for the determination of Ra-226.

**Fig. 3:**
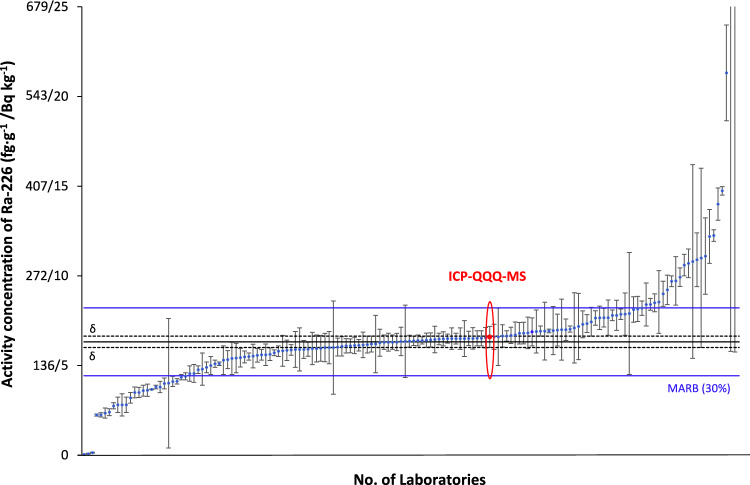
Results of Ra-226 determination in water sample by participants in IAEA World Wide and Almera Laboratories. Ra-226 reference value: 6.32 ± 0.32 Bq kg^-1^ (172 ± 9 fg g^-1^). ICP-QQQ-MS: 6.55 ± 0.57 Bq kg^-1^ (178 ± 15 fg g^-1^). MARB (minimum acceptable relative bias).

The reported result obtained with this direct method (Fig. 3) was 6.55 ± 0.57 Bq kg^-1^ (179 ± 16 fg g^-1^). The zeta (ζ)score, which provides a combined assessment of the reported value and the reported measurement uncertainty and thus the accuracy of the reported result, is 0.35, which means that the submitted result is acceptable, |ζ|≤ 2.

To our knowledge, all other laboratories used radiometric methods, thus this is the first time that an MS method (Agilent 8900 triple quadrupole mass spectrometer) has met the criteria for accuracy (trueness and precision) of a proficiency test for the quantification of Ra-226.

### Ra-226 in environmental water samples

The developed method was successfully applied for the determination of Ra-226 in various environmental water samples (Table S3), with measured activities ranging from 4.44 ± 1.30 to 209 ± 33 mBq kg⁻^1^ (0.12 ± 0.04 to 5.71 ± 0.89 fg g^-1^). The level of signal suppression at m/z = 226 varied between samples, from negligible to 35% suppression compared to the signal intensity of Ra-226 standard in 2% nitric acid solution, highlighting the complexity of different water matrices. In addition to hard water samples with elevated calcium and magnesium concentrations, significant signal suppression was also observed in soft water samples containing high levels of sodium or potassium. Despite this, the detection limits achieved across all samples remained below 0.19 fg g⁻^1^ (7 mBq kg⁻^1^). Moreover, Ra-226 was quantifiable in all measured samples at concentrations exceeding the detection limit specified by the EU Directive, confirming the suitability of the applied standard addition method.

A Spearman’s rank order correlation was used to assess the relation between chosen mayor and trace elements and Ra-226. A strong positive correlation was observed between Ca and Ra concentrations (ρ = 0.90, *p* = 0.002), as well as between Sr and Ra (ρ = 0.71, *p* = 0.0456), indicating that these alkaline earth metals likely share similar geochemical behaviour in natural waters. In contrast, no statistically significant correlation was found between Ba and Ra (ρ = 0.05, *p* = 0.9108), suggesting differing sources or mobility mechanisms. Ba concentration was similar in all analysed samples (except tap water) while concentrations of Ca, Sr and Ra varied.

Overall, the method proves to be robust and sensitive enough for use in routine monitoring. Additionally, it can be used in geochemical studies, especially within environments containing elevated levels of radium such as brines, thermal, or mineral waters. Moreover, the observed correlations with Ca and Sr suggest potential pathways for tracing Ra mobility and understanding geological influences on radionuclide distribution. The mehod can be implemented in environmental monitoring for periodic or continuous measurement and assessment of environmental parameters, such as pollution levels. Furthermore, alongside the regular analysis of chemical compounds using ICP-MS, it is possible to assess the combined presence of chemical toxicity and radiotoxicity in the same sample aliquot.

## Materials and methods

The sensitivity of ICP-QQQ-MS was evaluated prior method establishment. Tap water which contains common matrix elements was tested to evaluate spectral interferences on Ra-226 signal. A standard addition method was developed by spiking samples with Ra-226 to overcome possible matrix effect. The accuracy and precision of the method were assessed by analysing certified reference material and by participating in proficiency test exercise.

### Optimization of ICP-QQQ-MS and measurement procedure

#### Measurements with ICP-QQQ. instrument settings

Analyses were performed with an Agilent 8900 Series (Agilent Technologies, Japan) triple quadrupole inductively coupled plasma mass spectrometer ICP-QQQ-MS, which has an octopole collision-reaction cell positioned between two quadrupole mass filters (Q1 and Q2). The instrument was equipped with an autosampler (Agilent SPS-4, Agilent Technologies, Japan), a MicroMist nebulizer (0.4 mL min^−1^) and standard lenses and sample introduction system (x-lens, nickel sample and skimmer cones, and a Scott double pass (quartz) spray chamber). Manual tuning with 0.273 fg g^-1^ (10 Bq kg^-1^) Ra-226 standard solution in 2% nitric acid (Suprapur®) or acidified tap water was done for corrections of the torch axis, detector, plasma and lens voltage for maximum sensitivity. The instrument was used as a conventional ICP-Q-MS instrument, with the first quadrupole Q1 operated as mass filter, since this mode should provide the highest sensitivity^32,49^. The integration time was set to 10 or 30 s, depending on the concentration of Ra-226 in measured sample. One measurement consists of five replicates. The instrument operating conditions are summarized in the Supporting Information (Table S1). Overall time per sample was 4.5 min, consuming 1 mL of the sample.

### Sample preparation

The standard solutions of ^226^Ra (0.1—275 fg g^-1^/0.004—10.1 Bq kg^-1^) were prepared from a ^226^Ra certified standard solution (*c*_a_ = 368.7 Bq kg^-1^ ± 0.6%, reference date: 5th of June 2023) purchased from CMI (Czech Republic) in type I water obtained from a Direct-Q 5 system (Millipore, Watertown, MA, USA) and 2% (V/V) Suprapur® HNO_3_ (Sigma Aldrich, Germany). It was used for calibration and interference correction of ICP-MS optimization, and daily performance checks. Blank samples were prepared by acidifying Type I water with suprapur-grade nitric acid to a final concentration of 2% (v/v), and were measured in the same manner as the Ra-226 standard solutions.

### Testing for spectral interferences and matrix effect

Tap water was diluted or concentrated by evaporation to study matrix effect and possible spectral interferences on the Ra-226 signal in a typical water sample. Additionally, combination of 2 µg g^-1^ Sr and 50–100 ng g^-1^ of Ba as well as Pb up to 100 ng ^-1^ were tested for possible interferences on m/z 226.

Selected elements (Na, K Mg, Ca, Sr, Ba, Pb) were also determined in tap water. Surface water reference material SPS-SW1 (Spectrapure Standards, Manglerud, Norway) was used for the accuracy check.

### Sample preparation for standard addition method

Test solutions were prepared to evaluate the standard addition procedure. Two parallel samples were prepared with a known activity of Ra-226 in MiliQ or tap water. A parallel sample was spiked with an additional known (activity) concentration of Ra-226. Both samples were then diluted to same final volume, adjusting to 2% of nitric acid in the final solutions. After each 5 samples tap water spiked with CRM Ra-226 solution (0.273 fg g^-1^ (10 Bq kg^-1^)) was measured for quality control and to follow drift in the sensitivity (unintended change in ion beam intensity).

Proficiency test samples were provided by ALMERA-IAEA intercomparison scheme organized by IAEA Seibersdorf and Monaco Analytical laboratories.

The established method was used for Ra-226 determination in several environmental water samples where in addition major and trace elements concentrations were determined.

### Calculation

Activity of Ra-226 in unknown sample was determined from following equations:

1 $${s}_{sample}={k}_{a}\cdot {c}_{a}\cdot \frac{{m}_{01}}{{m}_{t1}}$$

2 $${s}_{spike}={k}_{a}\cdot ({c}_{a}\cdot \frac{{m}_{02}}{{m}_{t2}}+ {c}_{astd}\cdot \frac{{m}_{std}}{{m}_{t2}})$$

where *k*_a_ is the method’s sensitivity for the analyte (cps/pg g^-1^ (mBq g^-1^),

*m*_o1_ is mass of sample in unspiked solution (g),

*m*_o2_ is mass of sample in spiked solution (g),

*s*_sample_ is signal of unspiked solution subtracted for background signal, ${s}_{\mathrm{sample}}={s}_{\mathrm{G,sample}}-{s}_{\mathrm{BG}},$ (cps),

*s*_spike_ is signal of spiked solution subtracted for background signal, ${s}_{\mathrm{spike}}={s}_{\mathrm{G,spike}}-{s}_{\mathrm{BG}},$ (cps),

*c*_a_ is Ra-226 activity concentration of original sample, pg g^-1^ (mBq g^-1^) or fg g^-1^ (µBq g^-1^),

*c*_a*std*_ is Ra-226 activity concentration of spike, pg g^-1^ (mBq g^-1^) or fg g^-1^ (µBq g^-1^),

*m*_std_ is mass of the Ra-226 spike (g),

*m*_t1_- total mass of the unspiked solution (*m*_o1_ + *m* of added acid) (g),

*m*_t2_- total mass of the spiked solution (*m*_o2_ + *m* of added acid + *m*_std_) (g).

As long as *m*_std_ is small relative to *m*_o_, the effect of the spike’s matrix on the sample’s matrix is insignificant. Under these conditions the value of *k*_A_ is the same in both Eq. 1 and Eq. 2, and the expression of *c*_a_ is as follows:

3 $${c}_{a}=\frac{{s}_{\mathrm{sample}} \cdot {c}_{\mathrm{astd}} {\cdot w}_{\mathrm{std}}}{{s}_{\mathrm{spike}}\cdot {w}_{1} {- s}_{\mathrm{sample}} {\cdot w}_{2}}$$

where ${w}_{\mathrm{std}}$ is mass ratio of ${m}_{\mathrm{std}}/{m}_{t2}$, ${w}_{1}$ is mass ratio of ${m}_{\mathrm{o1}}/{m}_{t1}$, and ${w}_{2}$ is mass ratio of ${m}_{\mathrm{o2}}/{m}_{t2}$.

In the calculations mass was used instead of volume since all samples were weighed on an analytical balance (Mettler Toledo XPE205, USA) to obtain higher degree of accuracy and precision.

Equations used to validate the method and measurement uncertainty calculation^57^ are given in the Supporting Information (S4). The association of certain major and trace elements and Ra-226 was investigated using Spearman’s rank-order correlation.

## Supplementary Information

Supplementary Information.

## Data Availability

The datasets used and/or analysed during the current study are available from the corresponding author on reasonable request.

## References

[1] Paridaens, J. & Vanmarcke, H. Radium contamination of the banks of the river Laak as a consequence of the phosphate industry in Belgium. *J. Environ. Radioact.* **54**, 53–60 (2001). 11379074 10.1016/s0265-931x(00)00165-x

[2] United Nations Scientific Committe on the Effects of Atomic Radiation. Sources and effects of ionizing radiation volume I: source. *United Nations Sci. Comm. Eff.* **1**, 1–654 (2000).

[3] Garcia, D. & Matthews, T. Contaminated drinking water and its effect on cancer. *ACS ES T Water* **4**, 3340–3347 (2024).

[4] *Council Directive 2013/51/Euratom of 22 October 2013 laying down requirements for the protection of the health of the general public with regard to radioactive substances in water intended for human consumption*. (2013).

[5] *United States Environmental Protection Agency. National Primary Drinking Water Regulations, EPA 816-F-09-004*, (Accessed 21 February 2024). https://www.epa.gov/ground-water-and-drinking-water/national-primary-drinking-water-regulations#RADS. (2009).

[6] Lindsey, B. D., Cravotta, C. A., Szabo, Z., Belitz, K. & Stackelberg, P. Relation between road-salt application and increasing radium concentrations in a low-pH aquifer, Southern New Jersey. *ACS ES T Water* **1**, 2541–2547 (2021).

[7] International Atomic Energy Agency. the environmental behaviour of radium: Revised Edition. Environ. Behav. Radium Revis. Ed. 1–267. (2014).

[8] Al-Hamarneh, I. F. & Almasoud, F. I. A comparative study of different radiometric methodologies for the determination of 226Ra in water. *Nucl. Eng. Technol.* **50**, 159–164 (2018).

[9] Forte, M. et al. Validation of a method for measuring 226Ra in drinking waters by LSC. *Appl. Radiat. Isot.* **103**, 143–150 (2015). 26093366 10.1016/j.apradiso.2015.05.022

[10] Abbasi, A. A review of the analytical methodology to determine Radium-226 and Radium-228 in drinking waters. *Radiochim. Acta* **106**, 819–829 (2018).

[11] Jia, G. & Jia, J. Determination of radium isotopes in environmental samples by gamma spectrometry, liquid scintillation counting and alpha spectrometry: a review of analytical methodology. *J. Environ. Radioact.* **106**, 98–119 (2012). 22245211 10.1016/j.jenvrad.2011.12.003

[12] Chmielewska, I., Chalupnik, S., Wysocka, M. & Smolinski, A. Radium measurements in bottled natural mineral-, spring- and medicinal waters from Poland. *Water Resour. Ind.* **24**, 100133 (2020).

[13] Blanco, P., Lozano, J. C., Gómez Escobar, V. & Vera Tomé, F. A simple method for 210Pb determination in geological samples by liquid scintillation counting. *Appl. Radiat. Isot.* **60**, 83–88 (2004). 14687640 10.1016/j.apradiso.2003.09.011

[14] Canepa, C., Benzi, P. & Marabello, D. The dynamics of the detection of ^226^Ra in water by scintillation counting in nonequilibrium conditions. *J. Environ. Radioact.* **251–252**, 106970 (2022). 36027819 10.1016/j.jenvrad.2022.106970

[15] Yang, G. et al. Simple and sensitive determination of radium-226 in river water by single column-chromatographic separation coupled to SF-ICP-MS analysis in medium resolution mode. *J. Environ. Radioact.* **220–221**, 106305 (2020). 32560892 10.1016/j.jenvrad.2020.106305

[16] Lagacé, F., Foucher, D., Surette, C. & Clarisse, O. Quantification of ^226^Ra at environmental relevant levels in natural waters by ICP-MS: Optimization, validation and limitations of an extraction and preconcentration approach. *Talanta* **167**, 658–665 (2017). 28340775 10.1016/j.talanta.2017.02.031

[17] Thakur, P., Ward, A. L. & González-Delgado, A. M. Optimal methods for preparation, separation, and determination of radium isotopes in environmental and biological samples. *J. Environ. Radioact.* **228**, 106522 (2021). 33360557 10.1016/j.jenvrad.2020.106522

[18] Hou, X. & Roos, P. Critical comparison of radiometric and mass spectrometric methods for the determination of radionuclides in environmental, biological and nuclear waste samples. *Anal. Chim. Acta* **608**, 105–139 (2008). 18215644 10.1016/j.aca.2007.12.012

[19] Diez-Fernández, S., Isnard, H., Nonell, A., Bresson, C. & Chartier, F. Radionuclide analysis using collision–reaction cell ICP-MS technology: a review. *J. Anal. At. Spectrom.* **35**, 2793–2819 (2020).

[20] Tims, S. G., Hancock, G. J., Wacker, L. & Fifield, L. K. Measurements of Pu and Ra isotopes in soils and sediments by AMS. *Nucl. Instruments Methods Phys. Res. Sect B Beam Interact. Mater. Atoms* **223–224**, 796–801 (2004).

[21] Cohen, A. S. & O’Nions, R. K. Precise determination of femtogram quantities of radium by thermal ionization mass spectrometry. *Anal. Chem.* **63**, 2705–2708 (1991).

[22] Ghaleb, B., Pons-Branchu, E. & Deschamps, P. Improved method for radium extraction from environmental samples and its analysis by thermal ionization mass spectrometry. *J. Anal. At. Spectrom.* **19**, 906–910 (2004).

[23] Park, C. J., Oh, P. J., Kim, H. Y. & Lee, D. S. Determination of ^226^Ra in mineral waters by high-resolution inductively coupled plasma mass spectrometry after sample preparation by cation exchange. *J. Anal. At. Spectrom.* **14**, 223–227 (1999).

[24] Zoriy, M. V. et al. Determination of ^226^Ra at ultratrace level in mineral water samples by sector field inductively coupled plasma mass spectrometry. *J. Environ. Monit.* **7**, 514–518 (2005). 15877175 10.1039/b503011k

[25] Copia, L., Nisi, S., Plastino, W., Ciarletti, M. & Povinec, P. P. Low-level ^226^Ra determination in groundwater by SF-ICP-MS: optimization of separation and pre-concentration methods. *J. Anal. Sci. Technol.* **6**, 1–7 (2015).

[26] Larivière, D., Epov, V. N., Reiber, K. M., Cornett, R. J. & Evans, R. D. Micro-extraction procedures for the determination of Ra-226 in well waters by SF-ICP-MS. *Anal. Chim. Acta* **528**, 175–182 (2005).

[27] Varga, Z. Ultratrace-level radium-226 determination in seawater samples by isotope dilution inductively coupled plasma mass spectrometry. *Anal. Bioanal. Chem.* **390**, 511–519 (2008). 17593357 10.1007/s00216-007-1394-9

[28] Benkhedda, K., Larivière, D., Scott, S. & Evans, D. Hyphenation of flow injection on-line preconcentration and ICP-MS for the rapid determination of ^226^Ra in natural waters. *J. Anal. At. Spectrom.* **20**, 523–528 (2005).

[29] Sharabi, G., Lazar, B., Kolodny, Y., Teplyakov, N. & Halicz, L. High precision determination of ^228^Ra and ^228^Ra/^226^Ra isotope ratio in natural waters by MC-ICPMS. *Int. J. Mass Spectrom.* **294**, 112–115 (2010).

[30] Epov, V. N., Lariviere, D., Evans, R. D., Li, C. & Cornett, R. J. Direct determination of 226Ra in environmental matrices using collision cell inductively coupled plasma mass-spectrometry. *J. Radioanal. Nucl. Chem.* **256**, 53–60 (2003).

[31] Verlinde, M. et al. A new rapid protocol for ^226^Ra separation and preconcentration in natural water samples using molecular recognition technology for ICP-MS analysis. *J. Environ. Radioact.* **202**, 1–7 (2019). 30771696 10.1016/j.jenvrad.2019.02.003

[32] Dalencourt, C., Michaud, A., Habibi, A., Leblanc, A. & Larivière, D. Rapid, versatile and sensitive method for the quantification of radium in environmental samples through cationic extraction and inductively coupled plasma mass spectrometry. *J. Anal. At. Spectrom.* **33**, 1031–1040 (2018).

[33] Bonin, M., Larivière, D. & Povinec, P. P. Detection of radium at the attogram per gram level in copper by inductively coupled plasma mass spectrometry after cation-exchange chromatography. *Anal. Methods* **12**, 2272–2278 (2020).

[34] Boudias, M. et al. ^226^Ra and ^137^Cs determination by inductively coupled plasma mass spectrometry: state of the art and perspectives including sample pretreatment and separation steps. *J. Environ. Radioact.* **244–245**, 106812 (2022). 35042022 10.1016/j.jenvrad.2022.106812

[35] Warwick, P. E., Russell, B. C. & Croudace, I. W. Evaluation of inductively coupled plasma tandem mass spectrometry for radionuclide assay in nuclear waste characterisation. *J. Anal. At. Spectrom.* **34**, 1810–1821 (2019).

[36] Zheng, J., Sahoo, S. K. & Aono, T. Recent progress on mass spectrometric analysis of artificial radionuclides in environmental samples collected in Japan. *Nucl. Anal.* **1**, 100025 (2022).

[37] Zhu, Y. et al. Tandem quadrupole inductively coupled plasma mass spectrometry for the quantitative and isotopic analysis of rare earth elements and radionuclides. *J. Anal. At. Spectrom.*10.1039/D4JA00409D (2025).

[38] Roulier, M., Baya, P. A., Roberge, S. & Larivière, D. Comparison of radium-226 separation methods based on chromatographic and extraction resins for its determination by ICP-MS in drinking waters. *J. Mass Spectrom.* **59**, e5005 (2024). 38311471 10.1002/jms.5005

[39] Yamakawa, A., Yamashita, K., Makishima, A. & Nakamura, E. Chemical separation and mass spectrometry of Cr, Fe, Ni, Zn, and Cu in terrestrial and extraterrestrial materials using thermal ionization mass spectrometry. *Anal. Chem.* **81**, 9787–9794 (2009). 19886654 10.1021/ac901762a

[40] Arevalo, R., Ni, Z. & Danell, R. M. Mass spectrometry and planetary exploration: A brief review and future projection. *J. Mass Spectrom.* **55**, e4454 (2020). 31663201 10.1002/jms.4454 PMC7050511

[41] Ben Yaala, H., Fniter, R., Foucher, D. & Clarisse, O. Direct analysis of radium-226 in sediment by ICP-MS: an analytical challenge?. *J. Anal. At. Spectrom.* **34**, 1597–1605 (2019).

[42] Larivière, D., Brownell, D. K., Epov, V. N., Cornett, R. J. & Evans, R. D. Determination of ^226^Ra in sediments by ICP-MS: A comparative study of three sample preparation approaches. *J. Radioanal. Nucl. Chem.* **273**, 337–344 (2007).

[43] van Es, E. M., Russell, B. C., Ivanov, P. & Read, D. Development of a method for rapid analysis of Ra-226 in groundwater and discharge water samples by ICP-QQQ-MS. *Appl. Radiat. Isot.* **126**, 31–34 (2017). 28236556 10.1016/j.apradiso.2017.02.019

[44] Caroli, S., Forte, M., Nuccetelli, C., Rusconi, R. & Risica, S. A short review on radioactivity in drinking water as assessed by radiometric and inductively coupled plasma-mass spectrometry techniques. *Microchem. J.* **107**, 95–100 (2013).

[45] Zhang, T., Bain, D., Hammack, R. & Vidic, R. D. Analysis of radium-226 in high salinity wastewater from unconventional gas extraction by inductively coupled plasma-mass spectrometry. *Environ. Sci. Technol.* **49**, 2969–2976 (2015). 25642997 10.1021/es504656q

[46] Lariviere, D., Taylor, V. F., Evans, R. D. & Cornett, R. J. Radionuclide determination in environmental samples by inductively coupled plasma mass spectrometry. *Spectrochim. Acta Part B At. Spectrosc.* **61**, 877–904 (2006).

[47] Wang, W., Evans, R. D., Newman, K. & Toms, A. Automated separation and measurement of ^226^Ra and trace metals in freshwater, seawater and fracking water by online ion exchange chromatography coupled with ICP-MS. *Microchem. J.* **167**, 106321 (2021).

[48] Amr, M., Al-Meer, S., Taha, M., Othman, Z. A. & Alghoul, M. Ultratrace determination of Radium-226 in mineral water by collision/reaction cell inductively coupled plasma mass spectrometry. *Int. J. Phys. Sci.* **1**, 11–18 (2013).

[49] van Es, E. M. et al. The behaviour of ^226^Ra in high-volume environmental water samples on TK100 resin. *J. Radioanal. Nucl. Chem.* **312**, 105–110 (2017). 28366971 10.1007/s10967-017-5203-4 PMC5357471

[50] Joannon, S. & Pin, C. Ultra-trace determination of ^226^Ra in thermal waters by high sensitivity quadrupole ICP-mass spectrometry following selective extraction and concentration using radium-specific membrane disks. *J. Anal. At. Spectrom.* **16**, 32–36 (2003).

[51] Wærsted, F. M., Jensen, K. A., Reinoso-Maset, E. & Skipperud, L. High throughput, direct determination of 226 Ra in water and digested geological samples. *Anal. Chem.* **90**, 12246–12252 (2018). 30280885 10.1021/acs.analchem.8b03494

[52] Handbook of ICP-QQQ Applications using the Agilent 8800 and 8900. (Accessed 06 June 2024). https://www.spectroscopyonline.com/view/handbook-of-icp-qqq-applications-using-the-agilent-8800-and-8900.

[53] Dalencourt, C., Chabane, M. N., Tremblay-Cantin, J. C. & Larivière, D. A rapid sequential chromatographic separation of U- and Th-decay series radionuclides in water samples. *Talanta* **207**, 120282 (2020). 31594595 10.1016/j.talanta.2019.120282

[54] Bergamini, G. et al. Determination of ^226^Ra in drinking water samples by alpha spectrometry. *J. Radioanal. Nucl. Chem.* **307**, 829–834 (2016).

[55] Terrestrial Environmental Radiochemistry Laboratory, International Atomic Energy Agency. *IAEA-TERC-2023–01 World Wide Open Proficiency Test Exercise, Pie-charts, S-Shapes and Reported Results with Scores, the IAEA Terrestrial Environmental Radiochemistry Laboratory (TERC)*. (Accessed 13 September 2024). https://nucleus.iaea.org/sites/AnalyticalReferenceMaterials/Shared%20Documents/ProficiencyTests/IAEA-TERC-2023-01/IAEA-TERC-2023-01_summary_report.pdf. (2023).

[56] Terrestrial Environmental Radiochemistry Laboratory, International Atomic Energy Agency. *IAEA-TERC-2023-02 ALMERA Proficiency Test Exercise, Pie-charts, S-Shapes and Reported Results with Scores, the IAEA Terrestrial Environmental Radiochemistry Laboratory(TERC)*. (Accessed 13 September 2024). https://nucleus.iaea.org/sites/AnalyticalReferenceMaterials/Shared%20Documents/ProficiencyTests/IAEA-TERC-2023-02/IAEA-TERC-2023-02_summary_report.pdf. (2023).

[57] BIPM, IEC, IFCC, ILAC, ISO, IUPAC, IUPAP, and OIML. Evaluation of measurement data—Guide to the expression of uncertainty in measurement. Joint Committee for Guides in Metrology, JCGM 100. https://www.bipm.org/ documents/20126/2071204/JCGM_100_2008_E.pdf. (2008).

