# Supplementary material for: Direct method for Ra-226 analysis in water samples using ICP-QQQ-MS
Source: Sci Rep. 2026 Jan 22;16:3068. doi: 10.1038/s41598-025-18775-4 (PMC12830613; doi:10.1038/s41598-025-18775-4)
Supplement: Supplementary file 1 — Supplementary Information. [file 41598_2025_18775_MOESM1_ESM.docx]

**SUPPORTING INFORMATION**

**Direct method for Ra-226 analysis in water samples using ICP-QQQ-MS**

**Ivana Coha^1,2^, Marko Štrok^1^, Norbert Kavasi^3,4*^**

*1. Department of Environmental Sciences, Jožef Stefan Institute, 1000 Ljubljana, Slovenia*

*2. Laboratory for radioecology, Division for Marine and Environmental Research,*

*Ruđer Bošković Institute, 10000 Zagreb, Croatia*

*3. Regional Environmental Co-Creation Unit, Fukushima Institute for Research, Education and Innovation, 960-1295 Fukushima, Japan*

*4. Department of Biophysics and Radiation Biology, Semmelweis University, 1085 Budapest, Hungary*

e-mail: [kavasi.norbert.h4v@research.f-rei.go.jp](mailto:kavasi.norbert.h4v@research.f-rei.go.jp)

TABLE OF CONTENTS

Table S1, Optimized tuning conditions of ICP-MS S2

Table S2, Major and selected trace elements of tap water S3

Formulas used for method validation S3

Calculation of combined uncertainty S4

Detection limit determination in unknown samples S5

Example for Batch 2 S6

Table S3, Element composition of water samples and signal suppression S7

Table S1. Optimized tuning conditions for the quantification of radium by various instrumental conditions for Agilent 8900

| **ICP-MS** | **8900** |
| --- | --- |
| scan type/tune mode | single quad (MS) |
| ***Plasma Parametrs*** | |
| RF power (W) | 1550 |
| RF matching (V) | 1.8 |
| Sample depth (mm) | 4 |
| nebulizer gas (L min^-1^) | 1 |
| nebulizer pump (rps) | 0.1 |
| makeup gas (L min^-1^) | 0.1 |
| Lenses Parameters (V) | |
| Extract 1 | -18 |
| Extract 2 | -245 |
| Omega Bias | -105 |
| Omega Lens | 12 |
| Q1 Entrance | 4.5 |
| Q1 Exit | 5 |
| Cell Focus | -2.2 |
| Cell Entrance | -40 |
| Cell Exit | -60 |
| Deflect | 19 |
| Plate Bias | -55 |
| Q1 parameters | |
| Q1 Bias | -3 |
| Q1 Prefilter Bias | -40 |
| Q1 Postfilter Bias | -32.5 |
| Interface cones | |
| Sample cone Standard, Ni | |
| Skimmer cone Standard, Ni | |
| ***Acquisition Parameters*** | |
| m/z monitored (Q1) | 226 |
| Integration time (sec) | 30 |
| Number of replicates | 5 |
| Numbers of sweeps | 10 |

Table S2. Major and selected trace elements composition of analysed tap water

| Element | Concentration, ng·g^‑1^ |
| --- | --- |
| Na | 3 807±253 |
| K | 767±51 |
| Mg | 14 119±832 |
| Ca | 65 187±4 196 |
| Sr | 156±8.6 |
| Ba | 31.1±1.2 |
| Pb | 1.59±0.07 |

*standard uncertainty with a coverage factor of *k = 1*;

**Equations used for method validation**

*Trueness of the method* was evaluated by calculating relative bias according to the equation:

$\text{Bias }(\%) =\frac{x-X}{X}\cdot100$ (1)

Where *x* is measured value and *X* is assigned value.

*Zeta score (ζ)*, which considers both the uncertainty in the assigned value, *u*_X_, and the uncertainty of the tested method, *u*_x_, calculated according to the equation:

$\zeta=\frac{(x-X)}{\sqrt{{u_{x}}^{2}+{u_{X}}^{2}}}$ (2)

was also used to evaluate performance of the method.

*Combined uncertainty* is calculated as $u^{2}(y)=\sum_{i=1}^{n} u^{2}(x_{i})\cdot\left( {\partial y}/{\partial x_{i}} \right)^{2}$ ^1^ (3)

Particular uncertainty contributions are determined as relative variances $h^{2}(x_{i})={\left( {\partial y}/{\partial x_{i}} \right)^{2}\cdot u^{2}(x_{i})}/{u^{2}(y)}$, where *u*(*x_i_*) is the particular uncertainty of quantity *x_i_*, and *u*(*y)* is the combined uncertainty of the calculated value *y*. Particular uncertainty of quantity *x_i_* includes all sources of uncertainty that contribute to that quantity.

**Calculation of combined uncertainty of Ra-226 activity concentration**

$c_{a}=\frac{s_{\text{sample}} \cdot c_{\text{astd}} {\cdot w}_{\text{std}}}{s_{\text{spike}}\cdot w_{1} {- s}_{\text{sample}} {\cdot w}_{2}}$ (4)

where $w_{\text{STD}}$ is mass ratio of ${m_{\text{STD}}}/{m_{t2}}$, $w_{\text{1}}$ is mass ratio of ${m_{\text{o1}}}/{m_{t1}}$, and $w_{\text{2}}$ is mass ratio of ${m_{\text{o2}}}/{m_{t2}}$

Combined uncertainties of $w_{\text{i}}$ ratios are calculated as

$u^{2}(w_{i})=w^{2}\cdot\left( {u^{2}\left( m_{a} \right)}/{m_{a}^{2}}+{u^{2}(m_{b})}/{m_{b}^{2}} \right)$. (5)

Partial derivatives of quantities in the equation $c_{a}$ are

$\frac{\partial c_{a}}{\partial s_{\text{sample}}}=c_{a}^{2} \cdot\frac{s_{\text{spike}}\cdot w_{1}}{s_{\text{sample}}^{2}\cdot c_{\text{astd}}\cdot w_{\text{STD}}}$ (6)

$\frac{\partial c_{a}}{\partial c_{\text{astd}}}=\frac{c_{a}}{c_{\text{astd}}}$ (7)

$\frac{\partial c_{a}}{\partial s_{\text{spike}}}=-c_{a}^{2}\cdot\frac{w_{1}}{s_{\text{sample}}\cdot c_{\text{astd}}{\cdot w}_{\text{std}}}$ (8)

$\frac{\partial c_{a}}{\partial V_{\text{std}}}=\frac{c_{a}}{V_{\text{std}}}$ (9)

$\frac{\partial c_{a}}{\partial m_{\text{o1}}}=-c_{a}^{2} \cdot\frac{s_{\text{spike}}}{m_{T1} \cdot s_{\text{sample}}\cdot c_{\text{astd}} \cdot w_{\text{std}}}$ (10)

$\frac{\partial c_{a}}{\partial m_{\text{o2}}}=\frac{c_{a}^{2}}{m_{t2}\cdot c_{\text{astd}}{\cdot w}_{\text{std}}}=\frac{c_{a}^{2}}{c_{\text{astd}}\cdot m_{\text{std}}}$ (11)

$\frac{\partial c_{a}}{\partial m_{t1}}=c_{a}^{2} \cdot\frac{s_{\text{spike}} {\cdot m}_{\text{o1}}}{m_{t1}^{2}{\cdot s}_{\text{sample}}\cdot c_{\text{astd}}\cdot w_{\text{std}}}$ (12)

$\frac{\partial c_{a}}{\partial m_{t2}}={-c}_{a}^{2} \cdot\frac{s_{\text{spike}} {\cdot w}_{1}}{s_{\text{sample}}\cdot c_{\text{astd}} {\cdot m}_{\text{std}}}$ (13)

$s_{\text{sample}}=s_{\text{G,sample}}-s_{\text{BG}}$; $u^{2}\left( s_{\text{sample}} \right)=u^{2}\left( s_{\text{sample}} \right)+u^{2}\left( s_{\text{BG}} \right)$ (14)

**Detection limit determination in unknown samples**

The instrumental detection limit (IDL) was determined in 2 % nitric acid from background measurements (3), defined as average of blank counts plus 3 *SD*_blank_ and divided by the slope of a curve obtained from two points; the counts (*x*_1_) and the determined (activity) concentration of the measured unknown sample (*y*_1_) and the counts (*x*_2_) and total (activity) concentration in the spiked sample (sample + spike, *y*_2_).

$IDL=\frac{s_{BG}+3{SD}_{BG}}{k_{a}}$

$k_{a}=\frac{s_{\text{s}\text{pike}} {-s}_{\text{sample}}}{(c_{a} +c_{astd}) - c_{a}}$=$\frac{s_{\text{spike}}{-s}_{\text{sample}}}{c_{astd}}$

**Example for Batch 2**

| **No** | **Sample** |
| --- | --- |
|  | Background sample |
|  | Quality Control sample (QC) |
|  | Rinsing solution |
|  | Unspiked Sample1 |
|  | Spiked Sample1 |
|  | Rinsing solution |
|  | Unspiked Sample2 |
|  | Spiked Sample2 |
|  | Rinsing solution |
|  | Unspiked Sample3 |
|  | Spiked Sample3 |
|  | Rinsing solution |
|  | Unspiked Sample4 |
|  | Spiked Sample4 |
|  | Rinsing solution |
|  | Unspiked Sample5 |
|  | Spiked Sample5 |
|  | Rinsing solution |
|  | Quality Control sample (QC) |
|  | Rinsing solution |
| **No** | **Sample** |
|  | Background sample |
|  | Unspiked Sample6 |
|  | Spiked Sample6 |
|  | Rinsing solution |
|  | Unspiked Sample7 |
|  | Spiked Sample7 |
|  | Rinsing solution |
|  | Unspiked Sample8 |
|  | Spiked Sample8 |
|  | Rinsing solution |
|  | Unspiked Sample9 |
|  | Spiked Sample9 |
|  | Rinsing solution |
|  | Unspiked Sample10 |
|  | Spiked Sample10 |
|  | Rinsing solution |
|  | Quality Control sample (QC) |
|  | Rinsing solution |
|  | Background sample |

Table S3. Ra-226, major and selected trace elements composition of analysed environmental waters along with signal suppression on m/z 226 due to matrix effects

| **Element, concentration** | **Drain water 1** | **Drain water 2** | **Drain water 3** | **Drain water 4** | **Drain water 5** | **Drain water 6** | **Surface water** | **Well water** |
| --- | --- | --- | --- | --- | --- | --- | --- | --- |
| Na, ng·g^-1^ | 3174±183 | 5147±122 | 40569±2092 | 10406±275 | 2159±94 | 2056±59 | 17848.±987 | 129749±7065 |
| K, ng·g^-1^ | 1007±58.62 | 768.72±15.37 | 17487.03±197.49 | 7181.67±197.49 | 824.39±20.76 | 967.2±31 | 7782.71±747.11 | 609±22.23 |
| Mg, ng·g^-1^ | 7011.15±306 | 12428±411 | 39566±1446 | 20723±1008 | 6676±384 | 7873±280 | 3687±35 | 707±26 |
| Ca, ng·g^-1^ | 29672±823 | 26179±333 | 309233±8827 | 336217±13996 | 34685±476 | 92597±2788 | 7433±596 | 3775±356 |
| Sr, ng·g^-1^ | 119.1±4.3 | 127.3±2.7 | 2102±73 | 1483±64 | 118.6±2.1 | 223.3±11.0 | 120.9±3.8 | 40.14±2.49 |
| Ba, ng·g^-1^ | 45.93±2.33 | 63.64±1.37 | 53.01±1.76 | 37.17±1.41 | 64.33±2.26 | 57.62±1.69 | 87.89±1.91 | 15.07±0.45 |
| Pb, ng·g^-1^ | 0.95±0.04 | 0.46±0.02 | 0.38±0.01 | 0.49±0.03 | 0.58±0.03 | 0.91±0.02 | 6.07±0.04 | 0.03±0.002 |
| Ra, fg·g^-1^  (mBq·kg^-1^) | 0.33±0.08 (12.12±2.98) | < DL | 1.48±0.28  (54.08±10.3) | 5.71±0.89  (209±32.57) | 1.19±0.21  (43.59±7.56) | 2.27±0.32  (83.08±11.78) | 0.53±0.08  (19.38±2.75) | 0.12±0.04  (4.44±1.3) |
| Detection limit, fg·g^-1^ (mBq·kg^-1^) | 0.14 (5.3) | 0.13 (4.7) | 0.18 (6.5) | 0.19 (7.0) | 0.13 (4.9) | 0.15 (5.5) | 0.10 (3.6) | 0.10 (3.8) |
| Ra-226 signal intensity compared to 2 % nitric acid solution | 94% | 100% | 75% | 70% | 100% | 89% | 70% | 65% |
| Total Hardness | 103 | 117 | 935 | 925 | 114 | 264 | 34 | 12 |

*standard uncertainty with a coverage factor of *k = 1*.
